# Mitogenomics in Amanita, evolution, species delimitation and its utility at the species complex level

**DOI:** 10.1099/mgen.0.001792

**Published:** 2026-07-30

**Authors:** Christian Quintero-Corrales, Roberto Garibay-Orijel

**Affiliations:** 1 Departamento de Botánica, Instituto de Biología, Universidad Nacional Autónoma de México, Coyoacán, Ciudad de México, C.P. 04510, Mexico; 2 Posgrado en Ciencias Biológicas, Universidad Nacional Autónoma de México, Coyoacán, Ciudad de México, Mexico

**Keywords:** comparative genomics, cryptic species, fungi, introns, ORF, phylogeny

## Abstract

Mitochondrial genomes are among the most powerful sources for resolving evolutionary relationships in eukaryotes; however, their application in fungal phylogenetics remains underutilized. In this study, we employed comparative genomics and phylogenomics to evaluate the utility of the mitogenome within the genus *Amanita*, specifically at the species complex level. Our results demonstrate that mitochondrial coding regions provide robust species-level resolution, while structural features – including intron dynamics, synteny breaks and ORFs – reveal evolutionary trajectories often obscured by traditional nuclear markers. We identified highly variable gene synteny patterns and significant fluctuations in intron and ORF content, particularly within the *cob* and *cox1* loci. Notably, these mitogenomic features varied considerably even among closely related species within the same section. Our findings underscore the dual utility of the mitochondrial genome for both interspecific inference and the identification of new cryptic lineages within established species complexes. Furthermore, we report a significant mitonuclear discordance, suggesting complex evolutionary histories such as ancestral hybridization or incomplete lineage sorting. These results highlight the necessity of integrating mitogenomics into fungal systematics to improve our understanding of species boundaries and evolutionary dynamics.

**Impact Statement**

This study demonstrates that mitochondrial genomes are a superior yet underutilized tool for resolving complex evolutionary histories in fungi, surpassing the limitations of traditional nuclear barcodes. By uncovering lineages and significant mitonuclear discordance within the *Amanita* genus, our research highlights how mitogenomics can reveal evolutionary events, such as hybridization and incomplete lineage sorting. These findings provide a new framework for fungal systematics, advocating for the integration of comparative mitogenomics to accurately define species boundaries and understand the genomic mechanisms driving fungal evolution.

## Data Summary

Data are available in the NCBI with the accession number: *A*. ‘*cruentilemurum*’ (PX718926, PX718927, PX718928, ON528107, PX927100, PX927101, PX927102, PX927103 and PZ264387), *A*. *brunneolocularis* (PZ264246, PX686254, PX686255, PX686257, PX686258, PX686259, PX686256, PZ258327, PZ258328, PZ264247 and PX686253), *A*. *flavorubens* (PX718929), *A*. *rubescens* var. *alba* (PX718930), *A*. series *Mappae* sp. (PX718931) and *A*. *suballiacea* (PX718932).

## Introduction

*Amanita* species have been widely studied as model organisms for decades due to their relevance to forest ecology, cultural, gastronomic and medical importance [1–6]. Most species form ectomycorrhizal mutualisms with plant hosts in Fagaceae, Dipterocarpaceae, Fabaceae and Pinaceae [4, 5], while a few basal lineages are free-living, carbon-degrading species [4, 5]. Recent studies have demonstrated that the genus is monophyletic and is divided into the following: subgen. *Lepidella* (*Lepidella* section), subgen. *Amanita* (*Amanita*, *Amarrendiae*, *Caesareae* and *Vaginatae* sections) and subgen. *Amanitina* (*Amidella*, *Arenariae*, *Phalloideae*, Roanokenses, *Strobiliformes* and *Validae*) [7, 8]. However, despite the extensive background in taxonomy, phylogenetics and genomics research on *Amanita*, species delimitation remains unsolved in many species complexes [9].

Species complexes are composed of cryptic species that are phylogenetically distinct but morphologically indistinguishable or exhibit low phenotypic disparity; thus, they are often erroneously classified under the same binomial name [10–13]. This biological enigma has been detected in all branches of the web of life – including fungi, algae, plants, animals and protists – and represents a remarkable portion of the undiscovered biodiversity [12, 14]. Cryptic species contrast with species radiation, where phenotypic divergence outpaces genetic change. In cryptic species, genetic divergence occurs with minimal phenotypic variation [14]. Evolutionary hypotheses about the origin of species crypticity have been proposed: recent divergence time, phylogenetic niche conservatism, morphological stasis, convergence and parallelism [12–15].

Mitochondria are essential for eukaryotic life as they provide the energy required for critical cellular functions [16, 17]. Fungal mitogenomes contain varying numbers of protein-coding genes (PCGs), including components of the electron transport chain (cytochrome oxidase subunits 1 to 3, nicotinamide adenine dinucleotide dehydrogenase subunits 1 to 6, including 4L), ATP synthase subunits 6 to 9 and apocytochrome b. Additionally, fungal mitogenomes encode two rRNA subunits (large and small), ribosomal protein S3 (*rps3*) and a variable number of unique and shared tRNA genes [17, 18]. However, the gene composition, gene order, pseudogenization, duplication and gene loss or gain vary among species, including closely related taxa [5, 19]. The mitochondrial genome has proven to be a powerful molecular marker at various phylogenetic scales [20–22]. The use of the mitogenome offers advantages over traditional multilocus and nuclear approaches due to its multicopy nature per cell, small genome size (which facilitates sequencing and assembly), uniparental inheritance, low levels of recombination, high nucleotide substitution rate and high intraspecific genetic variation [19]. For instance, Li *et al*. [5] compared the mitogenome features of eight *Amanita* species, finding that closely related species may or may not share gene synteny and other features. The authors demonstrated the phylogenetic resolution of the mitogenome from the order to species level, obtaining significant support for all tree nodes. These findings suggest that mitogenome loci – due to the varying evolutionary pressures and uniparental inheritance – maintain a balance between genetic diversity and conserved positions, providing high-level phylogenetic signal [22, 23].

The aims of our research were (1) to compare the mitochondrial genomic rearrangement, gene synteny and intergenic variation among *Amanita* species and correlate it with taxonomical hierarchy; (2) to test the phylogenetic accuracy of the mitogenome for fungi as a complementary tool for species delimitation at different phylogenetic levels; and (3) to explore its usefulness, as a complementary tool, for species identification within species complexes. For this, we assembled 22 new mitogenomes for the *Amanita rubescens* species complex (hereafter *Rubescentes* species): *Amanita brunneolocularis*, *Amanita ‘cruentilemurum*’, *Amanita flavorubens* and *A. rubescens* var. *alba*, together with two additional assembled *Amanita* mitogenomes (*Amanita suballiacea* from *Phalloideae* section and *Amanita* series *Mappae* sp. from *Validae* section) previously sequenced by Quintero-Corrales *et al*. [9]. All assembled mitogenomes were compared among themselves, with those from Li *et al*. [5], as well as with additional mitochondrial genomes available in public databases (NCBI and Mycocosm). We found that mitochondrial size varies significantly among species (from 62 to 137 kbp) and that gene synteny patterns can differ even in closely related species. The mitogenome provided high support at different phylogenetic levels. At the species complex level, it showed species-specific intergenic rearrangements with shared and unique ORF and intron sequences.

## Methods

### Samples, assembly and comparisons

All samples used here were stored in the ‘Herbario Nacional de México’ (Mexican National Herbarium, MEXU) from the Instituto de Biología, UNAM (Table S1, available in the online Supplementary Material, for sample metadata). We used ~20 mg of dry stipe, frozen at −70 °C for 24 h and ground with TissueLyser LT at maximum revolutions for 2 min. The DNA was extracted following the protocol of the DNAeasy Plant Mini Kit (QIAGEN). The *Rubescentes* species used here were previously identified by Quintero-Corrales *et al*. [9] using multilocus nuclear phylogenies. The raw data were generated using Illumina NovoSeq 6000 and filtered and cleaned with FASTQC v0.11.9 (http://www.bioinformatics.babraham.ac.uk/projects/fastqc/) and Trimmomatic v0.40 [24]. The clean data were used for mitogenome assembly with GetOrganelle v1.7.7.1 [25], which uses internal mapping tools and the software’s fungal database (fungus_mt) with the flags -R (10) and varying k-mer sizes (21 to 121, with increments of 10). As two of the studied species had more samples than the others (*A. brunneolocularis*, *n*=11, and *A. ‘cruentilemurum’*, *n*=9), we used them for intraspecific variation analyses. Two additional *Amanita* mitogenomes *A. aff. suballiacea* (*Phalloideae* section) and *A*. series *Mappae* sp. were assembled using the same workflow as mentioned before. Both species data were acquired from Quintero-Corrales *et al*. [9].

The *A. rubescens* mitogenome [26] was downloaded from the Mycocosm portal [27]. Eight *Amanita* (*Amanita basii*, *Amanita muscaria*, *Amanita bisporigera*, *Amanita phalloides*, *Amanita brunnescens*, *Amanita pseudoporphyria*, *Amanita inopinata* and *Amanita thiersii*) species’ mitogenomes used by Li *et al*. [5] were also downloaded. Additionally, six other *Amanita* mitogenomes were acquired from the NCBI [*Amanita sinensis* (MZ647487.1), *Amanita oberwinkleriana* (PP409003.1), *Amanita pallidorosea* (PP978973.1), *Amanita rimosa* (PP978972.1), *Amanita eijii* (PP409005.1) and the unknown *A.* sp. (PP409004.1), hereafter tagged as *A*. series *Mappae* sp.].

All mitogenomes were annotated using GeSeq [28] from the Chlorobox [29, 30] portal using other *Amanita* mitogenomes as query in BLAT Reference Sequences (https://chlorobox.mpimp-golm.mpg.de/), tRNAscan-SE 2.0 portal (https://trna.ucsc.edu/tRNAscan-SE/index.html), MFannotator and MITOS2 [29, 30] with the genetic code 4 (mould, protozoan and coelenteral) and the Refseq89 Fungi reference dataset. The latter was used within the Galaxy portal [31] to identify homing endonucleases. The graphical maps were generated with OGDraw v1.3.1 [32] within the Chlorobox web service. All found introns were compared with the NCBI database using the blastn tool to identify homologous sequences and trace their distribution in fungal lineages. Also, ORFinder was used to identify ORF sequences and their potential functions (https://www.ncbi.nlm.nih.gov/orffinder/). The fourth genetic code, a minimum of 300 bp ORF length and the UniprotKB-Swiss-Prot and NCBI’s reference proteins databases were used. The 300 bp ORF length was chosen to discard random noise or false positive hits. We used both smartBLAST and blastp in the ORFinder webservice with the UniprotKB-Swiss-Prot and NCBI’s reference protein databases. The minimum threshold for considering a sequence homology was 50% identity and 50% query coverage together with significant e-values (<1×10⁻⁵).

Sequence alignments were performed with Mauve v2.4.0 [33] (https://darlinglab.org/mauve/mauve.html) for genomic rearrangement and structural and punctual mutations. The programme accomplished a multiple-genome alignment for comparative genomics and evolutionary dynamics. The algorithm detected homologous regions among sequences labelled as locally collinear blocks (LCB).

### Phylogenetic analyses and distance matrix

For evolutionary analysis, 17 loci were extracted from each mitogenome (14 core PCGs+*rps3*+2 rRNA genes). We used *Lyophyllum shimeji* (MH447975) from Li *et al*. [5] as an outgroup species. All introns were removed before the phylogenetic analysis. Each locus was independently aligned with the muscle algorithm and concatenated in mega v11 [34]. The IQ-TREE web service [35] was used for the model selection (with the corrected akaike information criterion) and the maximum likelihood (ML) analyses. The concatenated matrix of all PCG, *rps3* and the rRNA loci was 37,532 bp long with the following evolutionary models: TVM+F+I+G4 for *atp6*, *nad1* and *rps3*; HKY+F+G4 for *atp8*; K3Pu+F+I+G4 for *nad3*; TIM2+F+G4 for *nad4L*; and GTR+F+I+G4 for *atp9*, *cob*, *cox1-3*, *nad2*, *nad4-5*, *rnl* and *rns*. The per-partition models detailed above were used as independent subsets (unlinked partitions) for the ML analysis. Bayesian analyses were performed with MrBayes v3.2 [36] on the bioinformatic cluster of CONABIO, using 25 million iterations, unlinked loci, 25% burn-in, sampling every 1,000 and the GTR+I+G evolutionary model for each locus partition. Chain convergence (trace plot) and effective sample sizes were tested with Tracer v1.7 [37]. Then, the saved iterations and trees were combined with LogCombiner v2.6.7, and the consensus tree was generated with TreeAnnotator v2.6.3 from BEAST v2.5 [38]. Trees were visualized with FigTree v1.4.4 (http://tree.bio.ed.ac.uk/). A genetic distance matrix analysis was performed in mega v11 using the same species (40) used for the phylogenetic inference, setting 1,000 bootstraps, the Kimura-2-parameter (K2P) model and using the mould, protozoan and coelenterate genetic code. We chose the K2P model as the genetic distance model as it better fits with closely related species.

To evaluate the influence of geographic distance on mitogenome diversification, we performed Mantel tests using R packages vegan v2.6-19 [39] and geosphere v1.6-8 [40] in the RStudio IDE (Posit team, 2025, http://www.posit.co/). Genetic distances were calculated as previously described, but only with *A. cruentilemurum* and *A. brunneolocularis* samples (20), and geographic distances were derived from approximated geographic coordinates (GPS). Due to the presence of two distinct sympatric groups (*A. ‘cruentilemurum’* and *A. brunneolocularis),* we performed a partial Mantel test controlled for lineage identity (e.g., 0 for a species and 1 for the other species) as well as independent standard Mantel tests for each species to assess lineage-specific isolation-by-distance (IBD) patterns.

## Results

**Fig. 1.**
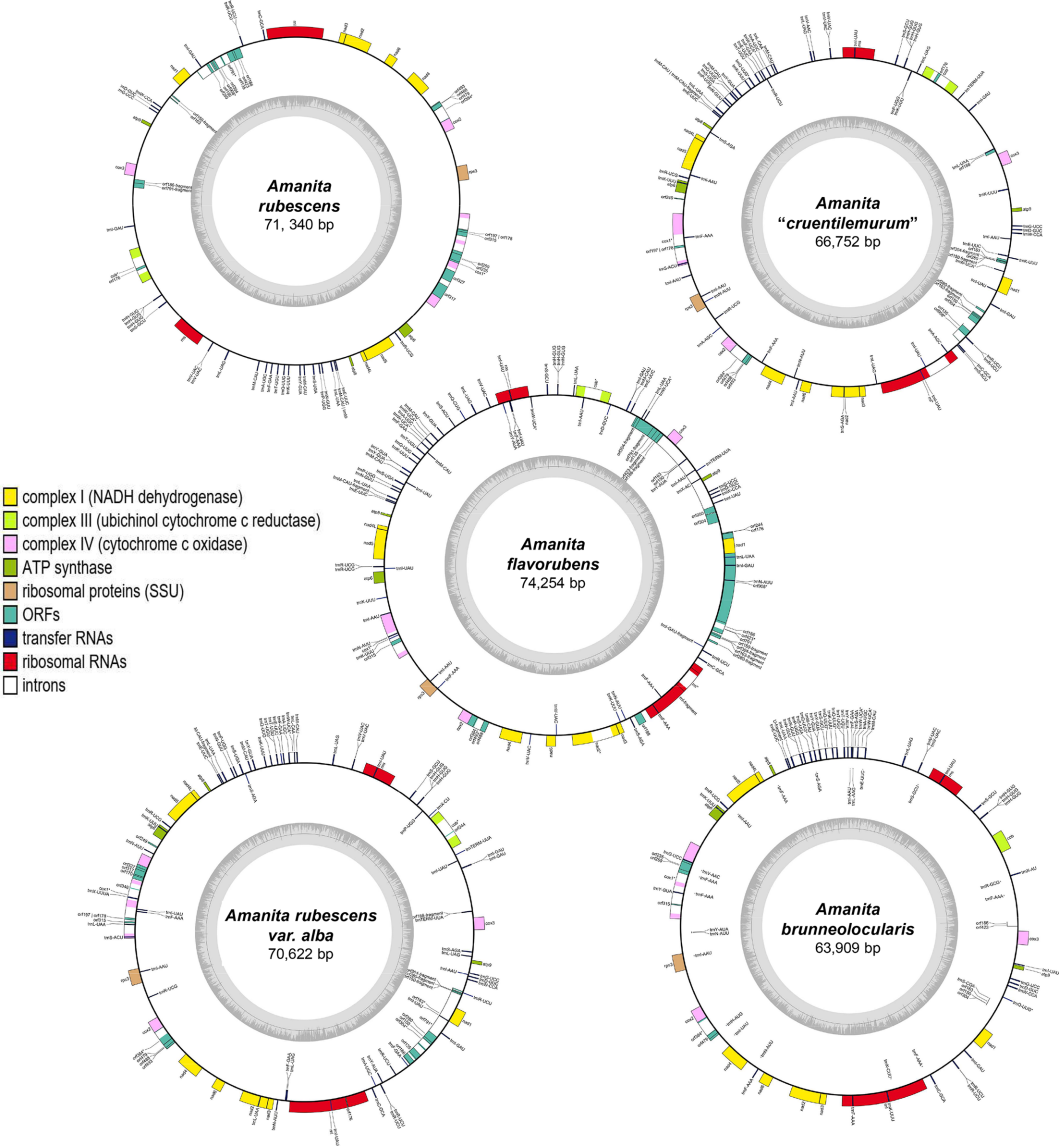
Mitochondrial maps of different *Amanita Rubescentes* series species. Different-coloured blocks in the circle maps represent each gene. The blocks outside the ring are in the forward gene direction, while the ones inside are in the reverse. We selected one representative of the *A. brunneolocularis* (GNHR433) and *A. ‘cruentilemurum’* (ART-2019-28) to illustrate their assembled mitogenomes (see Figs S2 and S4).

Twenty-two new mitogenomes were assembled for four different species from the *Rubescentes* series species: *A. brunneolocularis*, *A. ‘cruentilemurum*’, *A. flavorubens* and *A. rubescens* var. *alba* (Fig. 1 and Tables S2–S4, for gene order in *Rubescentes* species); we also included the *A. rubescens* mitogenomes obtained from Mycocosm. The lengths of the *Rubescentes* mitogenomes ranged from 63,868 to 74,254 bp, with GC ratios of 21.12 to 22.3.

All 14 PCGs, 1 *rps3* and 2 rRNA (*rnl* and *rns*) loci were found in all *Rubescentes* species (Tables S2–S4). Many intronic and non-intronic regions, ORFs, GIY-YIG and LAGLIDADG homing endonucleases were detected throughout the mitogenomes of the species. Most *Rubescentes* species showed an intron in the *cob* gene, except for *A. brunneolocularis*. For the *cox1* gene, species showed different intron patterns: *A. ‘cruentilemurum*’ and *A. flavorubens* had one intron, *A. brunneolocularis* had two and *A. rubescens* and *A. rubescens* var*. alba* had three introns. The intronic region in *rnl* and *cob* encodes for LAGLIDADG in those species where they are present.

### Evolutionary relationships of *Amanita* species

**Fig. 2.**
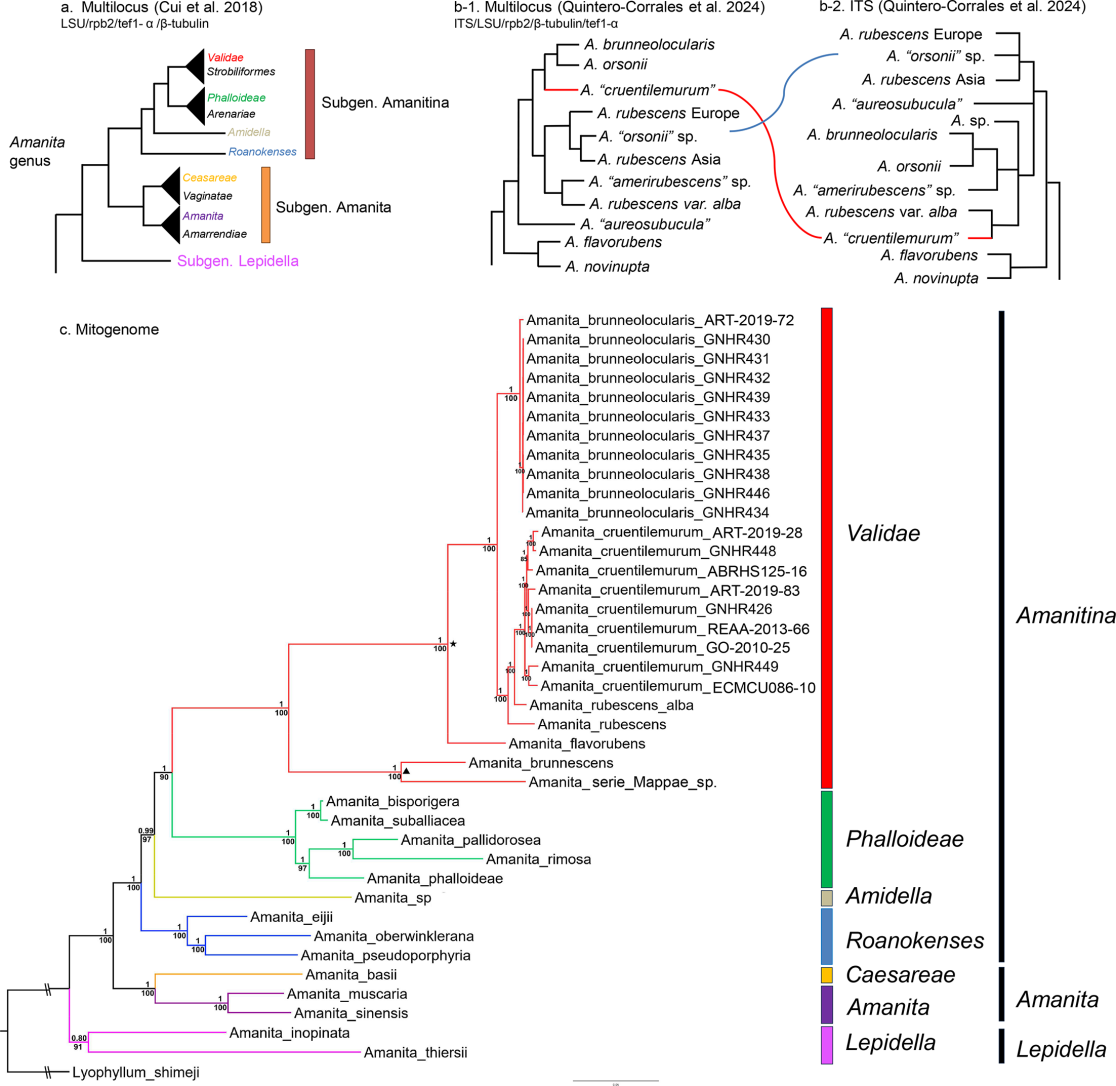
*Amanita* phylogenetic relationships based on different molecular markers. (a) Overview of the nuclear multilocus species tree of *Amanita* based on Cui *et al*. [7]. The authors used a concatenated matrix of the following loci: *LSU/rpb2/β-tubulin/tef1-α*. (b-1) Overview of the nuclear multilocus species tree of *Rubescentes* species (*Validae* section, red clade in (**a**) based on Quintero-Corrales *et al*. [9]. The authors used the following loci: *ITS/LSU/rpb2/β-tubulin/tef1-α*. (b-2) Overview of *ITS* species tree of *Rubescentes* species based on Quintero-Corrales *et al*. [9]. (**c**) Bayesian and ML mitogenome phylogeny analysis of the PCG, rps3 and the two rRNA loci of *Amanita*. Support values are shown as posterior probability (above) and bootstrap values (below). Coloured branches represent each *Amanita* section. The star mark shows the *Rubescentes* series node and the triangle icon the *Mappae* series node.

In both ML and Bayesian analyses, the topology of the genus *Amanita* was largely consistent with results obtained using traditional barcode genes (Fig. 2a, b). Seven of the 11 *Amanita* sections were recovered: *Phalloideae* (100% ML/1.0 pp), *Validae* (100% ML/1.0 pp), *Amidella* (97% ML/1.0 pp), *Roanokenses* (100% ML/1.0 pp), *Caesareae* (100% ML/1.0 pp), *Amanita* (100% ML/1.0 pp) and *Lepidella* (91% ML/0.80 pp, Fig. 2c). Within section *Validae*, two monophyletic and well-supported clades were resolved, corresponding to the two sister series within the section: *Rubescentes* (*A. flavorubens, A. brunneolocularis*, *A. rubescens*, *A. rubescens* var*. alba* and *A. ‘cruentilemurum*’, 100% ML/1.0 pp) and *Mappae* (*A. brunnescens* and *A*. series *Mappae* sp., 100% ML/1.0 pp). To identify *A*. series *Mappae* sp. as a member of the *Mappae* series, we used the *cox1* and *rns* loci as a query to the NCBI’s blastn tool: for the *cox1*, *A. brunnescens* and *A. porphyria* were the top hits, and for the *rns*, *A. citrina* (AF159072) and *Amanita aff. citrina* (HQ539873). To identify the second unknown *A.* sp. 1 as an *Amidella* species, we used partial *rns* and complete *rpb2* (obtained from the sample’s mitogenome), which species *Amanita curtipes*, *Amanita ponderosa*, *Amanita peckiana*, *Amanita volvata* and *Amanita fulvisquamea*, all from the *Amidella* section, were the upper matched with an identity percent of up to 95%.

The 17 concatenated loci distance matrix (14 core PCGs+*rps3*+2 rRNA genes) showed values from 0 to 0.224 (Table S5 for genetic distance): the outgroup (*L. shimeji*) had the highest mean distance values of 0.194 and the smallest of 0.010 (*A. brunneolocularis* GNHR446). The lowest value between species of different sections was 0.077 in *A*. *muscaria* and *A. basii* (from *Amanita* and *Caesareae* sections, respectively). Within each section, the values for species boundaries ranged from 0.0032 in *A. bisporigera* and *A. suballiacea* from the *Phalloideae* section to 0.057 in *Amanita oberwinklerana* and *Amanita pseudoporphyria* from the *Roanokenses* section.

The partial Mantel tests suggested a moderate correlation between geography and genetic distance between the species (*r*=0.388, *P*=0.001). However, when analysed independently, neither the *A. ‘cruentilemurum’* (*r*=0.027, *P*=0.436) nor the *A. brunneolocularis* (*r*=0.99, *P*=0.088) exhibited significant IBD.

### Synteny and mitogenomic rearrangements among *Amanita* and *Rubescentes* species

The mean mitogenome size across all *Amanita* species was 70.5 kbp with a mean G+C content of 22.49 mol%. Mitogenome features varied considerably at the sectional level (Fig. S1; Tables S6 and S7 for gene order in other *Amanita s*pecies and synteny, respectively). Section *Phalloideae* (five species) exhibited a mean size of 90.6 kbp and 25.24 mol% GC; *Roanokenses* (three species) averaged 54.2 kbp and 24.61 mol% G+C; *Caesareae* (one species) measured 37.3 kbp with 23.4 mol% G+C; *Amanita* (two species) showed a mean size of 65.6 kbp and 23.44 mol% G+C; *Lepidella* species averaged 98.5 kbp and 25.78 mol% G+C; *Amidella* (one species) measured 45.8 kbp and 22.43 mol% G+C; and *Validae* (five species) had a mean size of 67.4 kbp and 22.1 mol% G+C. The smallest mitogenome was found in *A. basii* (37 kbp; section *Caesareae*), while the largest was in *A. thiersii* (137 kbp, section *Lepidella*), both from Li *et al*. [5].

Nine different gene synteny groups were identified among the studied species. The most common synteny pattern was shared by eight species (40%; *A. muscaria*, *A. sinensis*, *A. basii*, *A. pseudoporphyria*, *A. eiji*, *A. oberwinklerana*, *A*. *aff*. *suballiacea* and *A. bisporigera*), followed by the *A. rubescens* species complex (five species, 25%) and two other species from section *Validae* (*A. brunnescens* and *A*. series *Mappae* sp.). The remaining six species (~30%) exhibited unique, non-shared synteny patterns: two in *Lepidella* species, one in *Amidella* and three distinct patterns within the remaining *Phalloideae* species. Some patterns were slightly different due to gene rearrangements, such as translocations or inversions. For instance, a comparison between the *A. rubescens* species complex and the most common pattern revealed differences at the *nad1* and *atp9* loci; in the *Rubescentes* species, the gene order was *nad1-atp9-cox3-cob-rrnS-atp8*, whereas the most common pattern was *nad1-cob-rrnS-atp9-cox3-atp8*. Similar minor variations were observed in the mitogenomes of *A. pallidorosea* and *A. rimosa* between the *nad3* and *cox3* loci (Fig. S1 and Table S7 for gene synteny).

Between 24 and 27 unique tRNA loci were detected across all *Amanita* mitogenomes, encoding all standard amino acids (Table S8 for tRNA distribution). Most species contained three copies of *trnM-CAU* and two to three copies of *trnI-GAU*, except for *A. pseudoporphyria*, *A. sinensis* and *A. inopinata*, which lacked *trnI-GAU. A. oberwinklerana* possessed two copies of both *trnR-UCU* and *trnF-GAA*, while *A. sinensis* was the only species with four copies of *trnK-UUU*. Additionally, unique tRNA genes were identified in several species: *trnN-AUU* in *A. rimosa*, *trnL-CAA* in *A. pallidorosea*, *trnT-GGU* in *A. muscaria* and *trnG-ACC* in both *A. inopinata* and *A. thiersii*. Furthermore, unusual DNA polymerase (*dpo*) and RNA polymerase (*rpo*) genes were located near the *nad1*, *nad3* and *cox3* loci in certain species (Tables S2–S6). Both *dpo* and *rpo* within the *Rubescentes* species complex showed low sequence similarity (<60%) to those in the NCBI database.

**Fig. 3.**
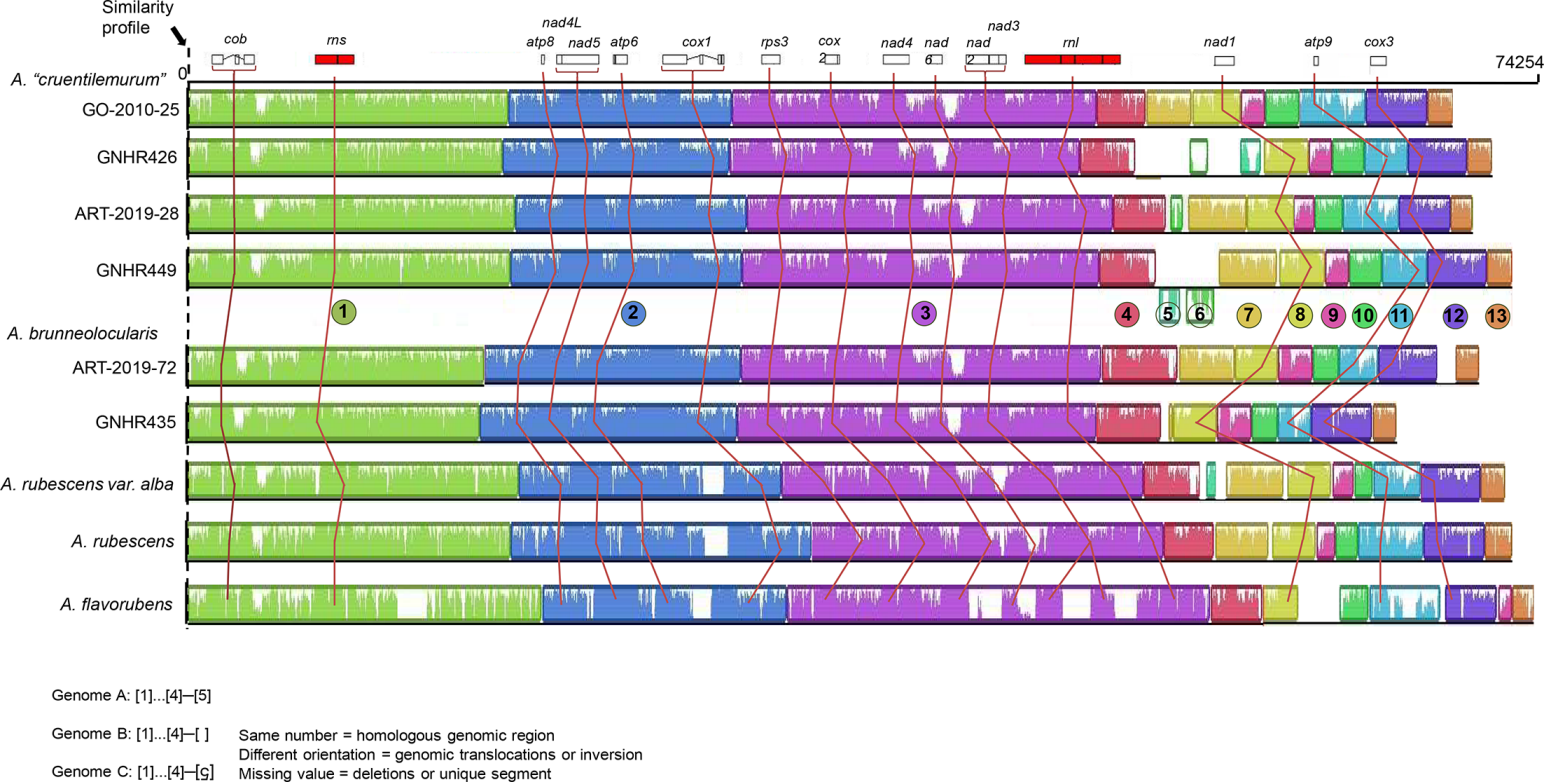
Comparison of mitochondrial genomes of *A. rubescens* species complex using Mauve v2.4.0. The horizontal axis in each sample or species diagram shows the mitogenome length, while the vertical axis shows a similarity profile at that specific site. Each rectangle above the image illustrates the PCG, *rps3* and the two rRNA loci position. Each coloured and numbered block (1 to 13) represents a homologous genomic region (LCB) shared among the different species. White spaces indicate unique DNA regions for that specific sample or species. Changes in the order or orientation (above/below the axis) indicate rearrangements. The vertical axis values are inverted for all elements below the line.

All *Rubescentes* species shared the same gene synteny configuration (Tables S2–S4, Fig. 3), and between the *rns* and *atp8* loci was identified as the tRNA-rich region. The mitogenome rearrangement analyses detected up to 13 shared LCBs among *Rubescentes* species, and the most variable region was observed between the *rnl* and *cob* genes (blocks #4 to #13, Fig. 3). We detected most of the mitochondrion’s ORF, *dpo* and *rpo* loci within this variable region (Table S9 for ORF information). However, some areas within each LCB showed low or no homology in some species. *A. ‘cruentilemurum’* sample GNHR449 was the only one with all 13 LCB. *A. flavorubens* had the largest mitogenome of all *Rubescentes* species (74.2 kbp) and had most of the non-shared DNA segments (white spaces within #1, #2, #3 and #11 and regions with no LCB blocks), it lacked #5 and #6 and there was no shared fragment between #8 and #10. *A. flavorubens* had at least one inverted/translocated region (#8 block), translocated blocks (#9) and LCB lost and reductions (#4, #7, #9 and #12). *A. rubescens* var. *alba* has a 70.6 kbp mitogenome with many unique fragments and shares part of #5. *A. brunneolocularis* showed two different LCB patterns (ART-2019-72 and all the other species). Most of the species’ samples had the smallest mitogenome size and almost lacked the LCB #7, except that ART-2019-72 had the full #7 block and a unique region between #12 and #13.

**Fig. 4.**
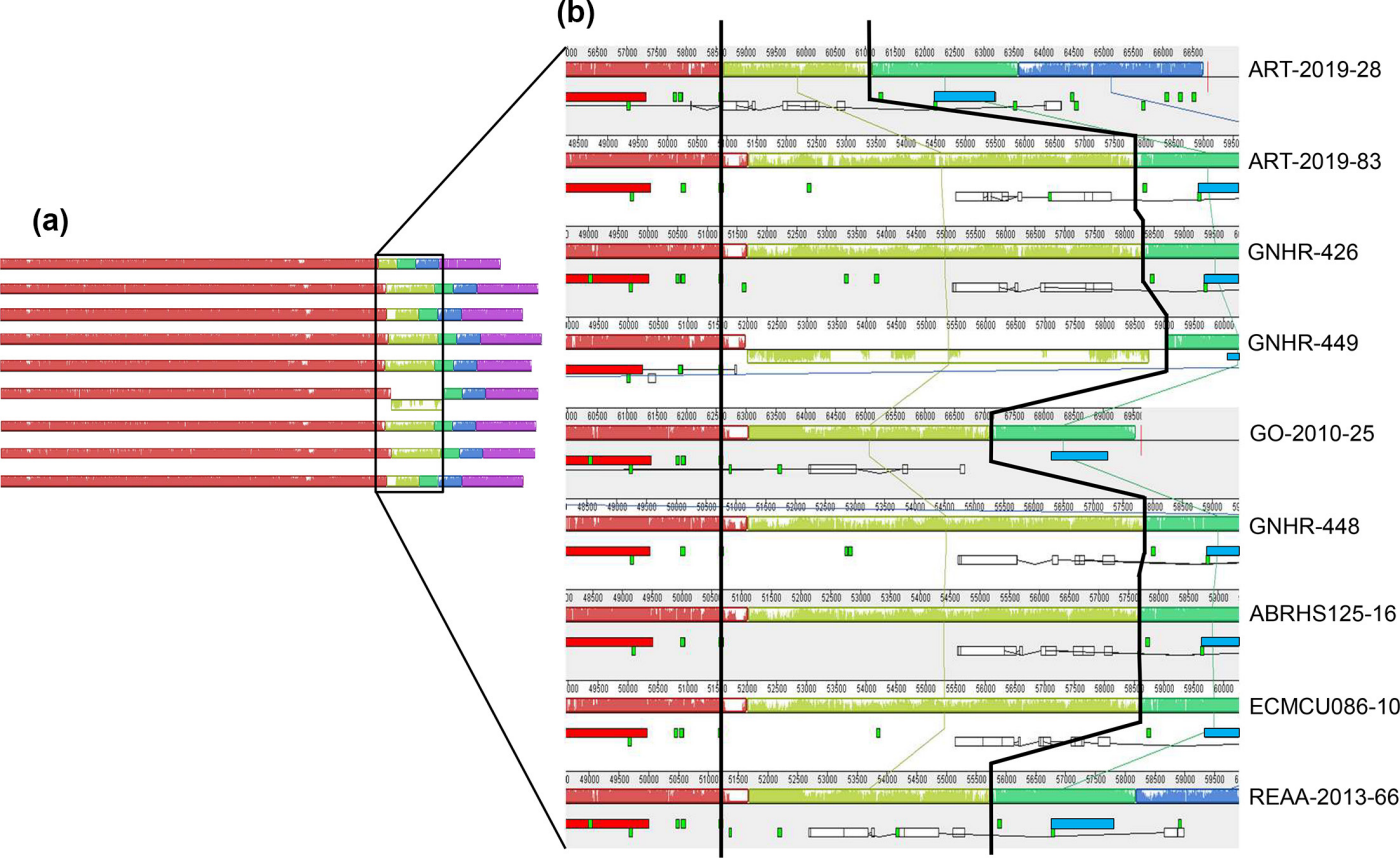
Mitochondrial intraspecific variation in *A. ‘cruentilemurum’* using Mauve v2.4.0. Each coloured region represents a shared homologous site among the different samples. (**a**) Whole-mitogenome comparison among samples (see Figs S2 and S3). (**b**) Zoom-in on an intergenic region among *A. ‘cruentilemurum*’ samples between *rnl* (red bar) and *nad1* (blue bar). The different-coloured areas are shared among samples, while the uncoloured regions are unique sequence fragments for each sample.

*A. ‘cruentilemurum*’ mitogenomes ranged in size from 66.7 to 71.6 kbp (Figs S2 and S3). All samples exhibited a genetic rearrangement of varying length between the *rnl* and *nad1* genes (Fig. 4), which accounted for most of the inter-sample variation. Five of nine *A. ‘cruentilemurum*’ samples displayed similar mitogenomic LCB and intergenic variation (Fig. S3). In contrast, sample GNHR-449 possessed an inverted LCB and unique variation, while three other samples (GO-2010-25, REAA-2013-66 and ART-2019-28) showed a reduction of one LCB. Specifically, it lacked a fragment of at least 4.4 kbp, whereas GO-2010-25 and REAA-2013-66 were missing a ~3 kbp region but exhibited a unique sequence of ~1.2 kbp. GNHR-449 was the only sample with an inversion, involving a ~7 kbp variation: ~3 kbp of this fragment were homologous to the sequence inverted in other samples, while the remaining sections consisted of unique variations containing unidentified ORFs.

In the intraspecific comparison of *A. brunneolocularis*, most mitogenomes were similar in size (~63 kbp) and shared the same LCB patterns, except for sample ART-2019-72, which was ~4.5 kbp larger (~68 kbp; Fig. S4). This size increase was attributed to three main regions: a 162 bp segment near the *cob* gene containing a newly acquired 86 bp ORF with no homology in the NCBI database; a second site of ~930 bp containing two unknown ORFs (522 bp and 63 bp); and a larger ~3,170 bp region between *rnl* and *nad1*. In all *A. brunneolocularis* samples, multiple distinct ORFs (up to 968 bp) were identified near the *nad1* gene, specifically located between the *trnR-UCU* and *trnI-GUA* loci.

ORFfinder identified up to 16 distinct ORF sequences (including both unique and shared sequences) among the *Rubescentes* species (Table S9). *A. flavorubens* exhibited the highest number of ORFs (16 total: 3 shared and 13 unique), ranging from 303 to 2,964 bp. *A. rubescens* var. *alba* possessed 12 ORFs (4 shared and 8 unique), ranging from 324 to 1,455 bp. Most *A. brunneolocularis* samples maintained the lowest number of ORFs (seven total: four shared and three unique; 324–1,188 bp), except for sample ART-2019-72, which harboured ten (four shared and six unique; 309–1,188 bp). *A*. ‘*cruentilemurum*’ showed varying numbers, ranging from 10 to 15 ORFs (5–11 shared and 0–9 unique; 309–2,940 bp). As previously noted, the genomic region between *rnl* and *nad1* is the primary hotspot for ORF gain and loss across all species, while the region with the highest concentration of ORFs was located between the *nad3* and *nad1* loci.

### Intron variability in *Amanita* mitochondrial genes

Intron content within mitochondrial genes varied across sections and species (Table S10 for the number of introns). A total of 137 intron sequences were identified across nine genes: *nad1* (5), *nad2* (2), *nad4* (1), *nad5* (5), *cob* (37), *rns* (6), *rnl* (16), *cox1* (60) and *cox2* (4). The distribution of *cox1* introns was non-uniform among species: section *Phalloideae* accounted for half of the identified introns (30), followed by *Validae* (19), with lower proportions in *Lepidella* (7) and *Amanita* (4). No *cox1* introns were detected in the remaining sections. Regarding *cob*, ~60% (22) of the introns were distributed within *Phalloideae*, while *A. thiersii* and section *Validae* accounted for 16% (6) and 15% (5), respectively. The mean number of introns per locus was ~1, excluding *cob* (2.6), *rnl* (1.8) and *cox1* (4).

Fifty of the 137 introns (36%) were not shared among *Amanita* species (Table S11 for the unique introns). We identified similar sequences in distantly related fungal lineages (15 different fungal families), including Tricholomataceae, Polyporaceae or Ganodermataceae. Half of these unique introns were found in the *cox* locus (27), followed by *rnl* (9) and *cob* (7); the remaining introns were from *nad1* (1), *nad2* (2), *nad4* (1), *nad5* (2) and *cox2* (1). Twenty-eight percent of these 50 unique introns (14) had no similarity match in the NCBI database, of which nine were from the *cox1* locus. The largest unique intron was intron #6 of *cox1* of 3,760 bp in *A. thiersii*, while the smallest was 59 bp long from the cox1 intron #5 from *A. pallidorosea*. The mean size of all unique introns was 1,295 bp.

*Validae* was the second section with the highest number of introns within its mitochondrial genes (31), most of which were in *cox1*, *rnl* and *cob*. Most of the genes’ introns were shared among other *Validae* species and some of them with other *Amanita* species. *A*. series *Mappae* sp. showed the highest number of introns (eight). Five of them were unique introns: four in *cox1*, two with no database hit and two with similar sequences to those of *Ganoderma* spp. The *cob*’s intron was also found in *Fomitiporia mediterranea*, the pathogen of grape fungi (Table S11). Two of the four *A. brunnescens* introns were unique, while the remaining ones were shared with *A.* series *Mappae* sp. (Table S11).

**Fig. 5.**
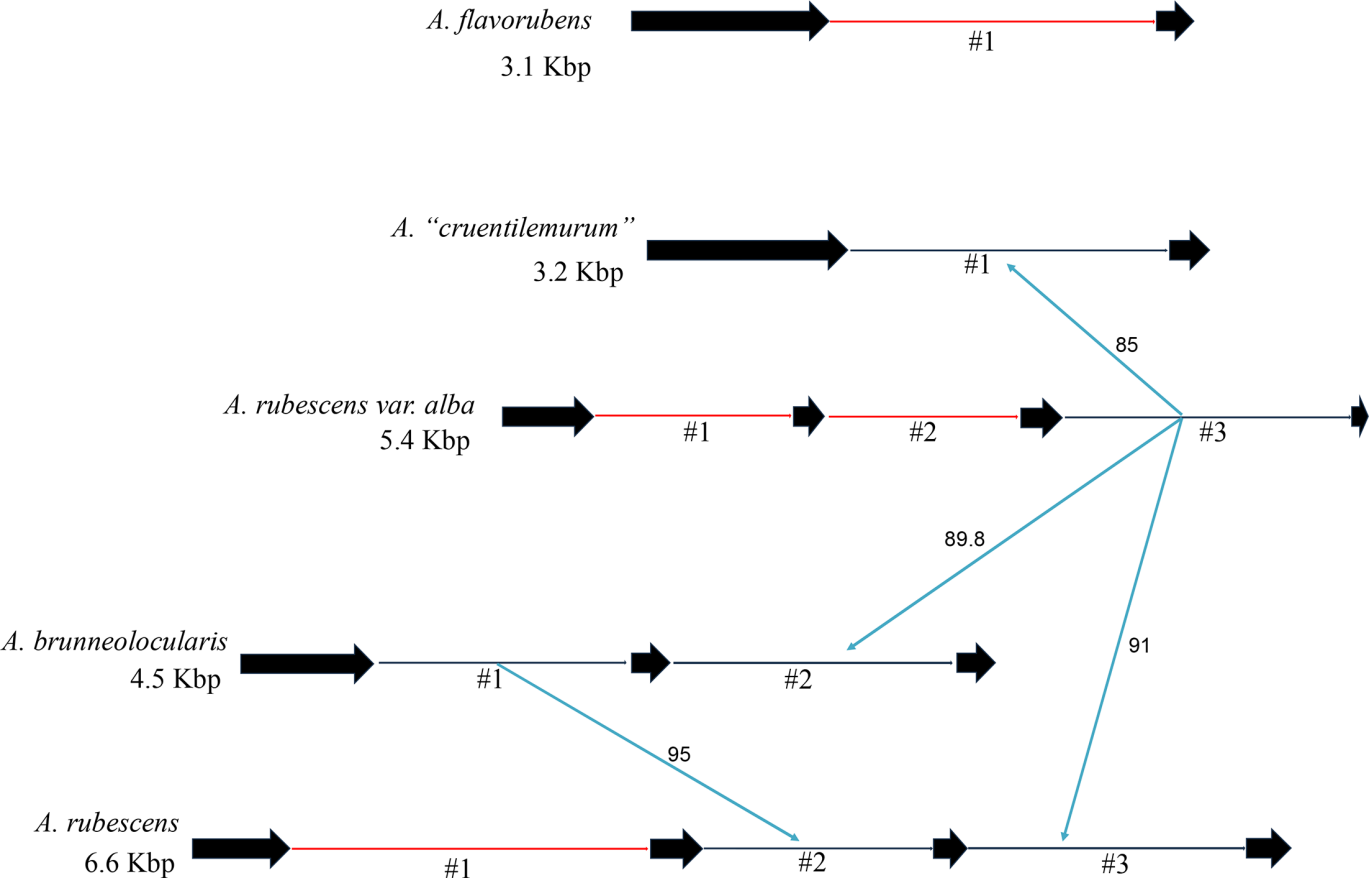
*Cox1* intron comparison among *Amanita Rubescentes* series species. The black arrows show exons, and thin lines indicate introns. The blue arrows connect shared introns among species, while the red lines indicate unique introns for that species. The full size of the *cox1* locus is shown below the species name.

Most *Rubescentes* species have introns in the *cox1*, *cob* and *rnl* genes. Four of the five species have a ~1,200 bp intron within the *cob* gene that is very similar among them. *A. flavorubens* was the only species with an intron of ~1,400 bp within the *nad2* locus; *A. flavorubens* and *A. ‘cruentilemurum*’ have introns in *rnl* loci of 1.1 and 1.5 kbp, respectively, but the remaining species did not (see discussion section). Finally, the *cox1* locus has up to three introns within it: *A. flavorubens* and *A. ‘cruentilemurum*’ had a single intron, *A. brunneolocularis* had two and *A. rubescens* and *A. rubescens* var. *alba* had three (Fig. 5). We compared the intron sequences among the different species and in the NCBI database to calculate their similarity. The *A. ‘cruentilemurum*’ single intron showed high similarity with all *A. brunneolocularis* intron2, *A. rubescens* intron3 and *A. rubescens* var*. alba* intron3. The first intron of *A. brunneolocularis* had 95% similarity with the second of *A. rubescens*. The remaining introns (*A. flavorubens* unique intron, the first and second from *A. rubescens* var. *alba* and the first from *A. rubescens*) lacked similar sequences to other *Amanita* species or the NCBI database (Table S11).

## Discussion

*Amanita* species have traditionally been identified based on morphological traits and a limited number of barcode loci [7]. However, the combination of sparse molecular data and restricted morphological characters, often coupled with inadequate sampling, has hindered the resolution of species complexes [9, 41]. In this study, we evaluated the efficacy of the mitogenome as a complementary marker to resolve evolutionary relationships and uncover candidate lineages across different phylogenetic scales. While previous studies morphologically assigned our *Rubescentes* specimens to the *A. rubescens* species complex, Quintero-Corrales *et al*. [9] utilized a five-locus concatenated matrix to demonstrate the presence of five distinct species: *A. rubescens*, *A. brunneolocularis*, *A. ‘cruentilemurum’*, *A. flavorubens* and *A. rubescens* var. *alba*. Our results demonstrate that the mitogenome provides robust evolutionary resolution across different phylogenetic scales, yielding higher support values than traditional markers and revealing previously unknown lineages within species complexes. Consequently, mitogenomic data represent a powerful complementary tool for systematics and taxonomy, significantly improving phylogenetic inference and uncovering candidate lineages that can be further evaluated for species delimitation.

### Mitochondrial evolution within the *A. rubescens* species complex

When utilizing the concatenated mitochondrial gene matrix (comprising PCGs, *rps3* and the two rRNA loci), we recovered a topological pattern consistent with the species tree based on nuclear genes [9], but with consistently higher support values at both the inter- and intraspecific levels. Both coding and non-coding regions exhibited evolutionary signatures of speciation within the species complex; in this group, the mitochondrial genome structure varied among species, despite maintaining conserved gene synteny. All *Rubescentes* mitogenomes displayed distinct genomic features, such as intergenic variation and total sizes ranging from 63 to 74 kbp (Fig. 1).

At the intra-species level, all *A. brunneolocularis* samples showed low genetic variation and similar mitogenome features, clustering in a phylogenetic polytomy. This could be explained by the close geographic origin of the samples (two localities ~50 km apart). The remaining *A. brunneolocularis* mitogenome sample is from Chiapas state, Mexico (ART-2019-72), which showed a unique ~4 kbp fragment but shared near-identical mitogenomic features despite being ~1,000 km away from the other *A. brunneolocularis* samples (Fig. S4). We had broader sampling for *A. ‘cruentilemurum*’ in Mexico and Guatemala. All samples showed intergenic variation near *cob*, *cox1*, *cox3* and the tRNA islands between *atp8* and *rns* together with the highly diverse region with genomic rearrangements between *rnl* and *nad1* (Figs 3, 4, S3 and S4). Among them, five of nine samples presented similar mitogenome features despite their geographic distance (from the same locality to up to ~900 km). In contrast, most of the remaining samples with different types of variation (deletion, *de novo* or inversion) came from closer localities, ranging from 12 to ~90 km, except for one sample from Guatemala, which is ~1,000 km away. The IBD analysis confirmed that geographic distance is not a primary driver of mitogenomic evolution in either species. The lack of a significant correlation (*P*>0.05 for both lineages) suggests that the observed structural rearrangements are driven by internal genomic mechanisms – such as the proliferation of introns – rather than geographic isolation. Our findings indicate that the two species exhibit contrasting patterns: *A. brunneolocularis* displayed lower genetic variation and fewer genomic rearrangements (suggesting relative genomic stasis) compared to *A*. ‘*cruentilemurum*’.

The multilocus analyses (ribosomal and three nuclear genes, Fig. 2b) from Quintero-Corrales *et al*. [9] showed the *Rubescentes* species evolutionary history. We showed that the mitogenome produced a similar phylogenetic hypothesis to those from the nuclear multilocus analysis: *A. flavorubens* as the most external taxon of the series, *A. brunneolocularis* in an individual cluster and *A. rubescens* within the American clade of *Rubescentes*. However, the placement of *A. ‘cruentilemurum’* by the mitogenome contradicts the nuclear phylogeny; here, it groups with *A. rubescens* var*. alba*, a tree topology consistent with the *ITS* outcomes. This phenomenon is called mitonuclear discordance and occurs when we observe dissimilar phylogenetic topologies across different genetic compartments (such as nuclear vs mitochondrial) [41]. This anomalous phylogenetic position could result from various evolutionary processes, such as incomplete lineage sorting (ILS), gene flow/hybridization among species, organellar capture, fluctuating demographic scenarios or selection pressures [41–44].

We propose two plausible but untested evolutionary scenarios to explain this discordance: first, a hybridization event with an ancestor of *A. rubescens* var. *alba*. Both species are sympatric, with overlapping geographical ranges spanning from Central and Southern Mexico to Eastern Canada and the USA [9], which may have facilitated secondary contact following their divergence, leading to mitochondrial capture. Under a mitochondrial capture scenario, the mitogenome phylogeny clusters with the donor species, while the nuclear phylogeny reflects the parental lineage [43]. Second, *A*. ‘*cruentilemurum*’ may retain ancestral polymorphisms that have persisted since divergence. In rapidly diverging lineages, characterized by short internal branches, such as in the *Rubescentes* species complex (Fig. 2c), the time elapsed since speciation may have been insufficient for complete lineage sorting, despite the typically small effective population size of mitochondrial genomes [42, 44, 45]. However, as with the current data, we could not test these events; whole-genome nuclear SNP extraction and analysis would be required to trace potential ancestral gene flow (using D-statistics, such as ABBA-BABA [46] tests or coalescent approaches, in SNAPP [47]) and shared polymorphisms (admixture [48]), which would be necessary to validate and distinguish between them.

The phylogenetic analyses revealed that the *A*. ‘*cruentilemurum’* clade exhibited multiple internal groups in all trees (Fig. 2), but only the mitogenome phylogeny showed significant support values. It was expected that genetic values among species from different sections would show higher genetic distance (Table S5). Within the sections, we found that the genetic distances among species ranged from 0.032 in *Phalloideae* section to 0.110 in *Validae* and *Lepidella*. However, a value of 0.0032 was observed between two different species from *Phalloideae: A. suballiacea* and *A. bisporigera*. These values were reflected in the species branch length and their phylogenetic closeness in the species trees. For instance, *A. suballiacea* and *A. bisporigera* have shorter branches than those of other species from the same section. For *A. ‘cruentilemurum’* clade, both the distance matrix and phylogenetic tree supported five candidate groups: (1) GNHR448 and ART-2019-28 (1 PP/100 bootstraps); (2) ABRHS125-16 (1 PP/85 bootstraps); (3) ART-2019-83 (1 PP/100 bootstraps); (4) GNHR426, GO-2010-25 and REAA-2013-66 (1 PP/100 bootstraps); and (5) ECMCU086-10 and GNHR449 (1 PP/100 bootstraps)(Fig. 2c). The genetic distance observed among these cryptic species has similar or greater values than those observed between *A. suballiacea* and *A. bisporigera*, ranging between 0.0032 and 0.0070 (Table S5). Therefore, it is likely that we could identify the hotspot of diversity where a potential new species complex lineage is within *A. ‘cruentilemurum’*, with a wide distribution from Central America to North America (Mexico, USA and Canada). A more exhaustive sampling effort throughout those locations, using micro- and macroscopic protocols together with nuclear information analyses, would be necessary to correctly identify whether these species are potentially new ones.

### Mitogenome gene rearrangements and synteny in *Amanita*

We identified diverse mitochondrial gene synteny among species within the same genus and across sections. Our results showed that mitochondrial rearrangements in *Amanita* species were not correlated with species lifestyles (i.e. mutualistic or saprophytic) or taxonomic section. For instance, both *Lepidella* species are asymbiotic but do not share gene rearrangements or mitogenome size (59 and 137 kbp). The remaining species form ectomycorrhizal symbiosis, and most of them shared gene rearrangements within the same section, but others did not, as observed in *Phalloideae* and *Validae*. Our results suggest that the potential ancestral mitogenome synteny pattern could be *Amanita–Caesareae–Roanokenses–Phalloideae*, as it is fully shared among different phylogenetic species (8 of 21 species, 38%) or partially as with *A. rubescens* species complex (5 species, 23%), which, combined, would be 13 species sharing a similar gene synteny (61% of the species). However, this statement is weakened by *Lepidella*’s species (*A. inopinata* and *A. thiersii*), which constitute the most distal branch of all *Amanita* and show different gene synteny patterns. It is necessary to increase sampling of *Amanita* mitochondrial genomes to resolve this, mainly from those sections with no assembled mitogenome, such as *Vaginatae*, *Amarrendiae* (both from subgenus Amanita), *Strobiliformes*, *Arenariae* (from subgenus Amanitina) and *Lepidella*. Furthermore, these new sampling efforts could explore if the synteny patterns could be a subgenus effect, as species from the *Caesareae* and *Amanita* sections from subgenus *Amanita* shared the same gene patterns with the most external branch of subgenus *Amanitina*, *Roanokenses*, while the remaining sections of the subgenus, *Amidella*, *Phalloideae* and *Validae*, showed the most variable gene patterns.

Here, we found that the intergenic hotspot in the *Rubescentes* species was located between the *rnl* and *nad1*, where most of the ORF diversity and unique DNA regions are. This variation in synteny, tRNA composition, and ORF content was primarily driven by the presence of introns and their associated homing endonuclease genes (HEGs), other transposable elements (such as dpo), tRNA rearrangements and mitochondrial recombination. Similar variations in gene synteny, ORFs and introns have been reported across diverse fungal taxa, regardless of their phylogenetic distance [49–52].

### Intron dynamics in the *Amanita* mitogenome

Introns in mitochondrial genes among *Rubescentes* species show interesting variation patterns. For instance, the loss of the cob locus intron in *A. brunneolocularis* and the gain of a 1.4 kbp intron in the *nad2* locus in *A. flavorubens*, with no homology. *A. flavorubens* and *A. ‘cruentilemurum*’ showed an intron in the *rnl* locus of 1.1 kbp and 1.5 kbp, respectively, but were not detected in *A. brunneolocularis* and *A. rubescens* var. *alba*. Among all other *Amanita* species, the *rnl* locus size ranges from 3.3 kbp to 3.7 kbp; however, in *A. brunneolocularis* and *A. rubescens* var. *alba,* it was ~5 kbp. Thus, it is likely that *A. brunneolocularis* and *A. rubescens* var. *alba* had an intron that the mapping tool did not detect (Fig. S5).

Our analysis revealed striking variation in *cox1* intron dynamics across the *Amanita* species studied. *A. flavorubens* and *A. ‘cruentilemurum*’ each possessed a single intron, *A. brunneolocularis* contained two, while *A. rubescens* and *A. rubescens* var. *alba* each had three introns. Surprisingly, the observed intron homology patterns contradicted our phylogenetic expectations. Given their close evolutionary relationships, we initially hypothesized that all *Rubescentes* species would share introns through vertical inheritance, with *A. flavorubens* (occupying the most external phylogenetic position) showing the lowest sequence similarity. We anticipated similar patterns for the second and third introns in the *cox1* gene. However, our findings revealed unexpected complexity: the single intron of *A. flavorubens*, along with the first and second introns of *A. rubescens* var*. alba* and the first intron of *A. rubescens*, showed no detectable similarity to any *Amanita* species or sequences in the NCBI database.

Intron variation has been widely documented among closely related species in other fungal groups. For instance, Zhang *et al*. [17] and Zhang *et al*. [53] analysed mitochondrial evolution in nematode-trapping fungi and worldwide *Agaricus bisporus* strains, respectively. Both studies reported conserved gene synteny, yet identified mitogenome size variation driven primarily by the presence or absence of introns. These researchers examined intron dynamics across different phylogenetic (interspecific and intraspecific) and biogeographic scales, demonstrating that mitogenomic variation occurs mainly within intergenic regions. This variation includes the number and size of introns, ORFs, plasmid-like sequences and homing endonucleases [54]. It is well established that introns and HEGs co-evolve, contributing significantly to mitochondrial genome expansion and structural architecture. Phylogenetic and evolutionary insights from 30 newly assembled mitochondrial genomes revealed that co-evolution of introns and HEGs shapes mitogenome size variation [50, 55], a pattern consistent with our observations in *Amanita*.

Megarioti and Kouvelis [50] analysed the evolutionary dynamics of introns and their homing endonucleases, among different orders from seven fungal phyla. The authors showed that, similar to our findings and all previously mentioned research, both *cob* and *cox1*, followed by the *rnl* locus, dominate the intron content and dynamics (high introns gain and loss) compared to the other mitochondrial genes, acting as hotspots for mobile genetic elements such introns. Pérez *et al*. [56] proposed that this evolutionary dynamic could be related to regulatory mechanisms in response to environmental conditions, compromising mitochondrial function.

Intron evolution is highly dynamic, driven by gains through homing endonuclease-mediated integration and losses via genomic deletion [49, 51]. These processes occur across species boundaries – as seen in the *Rubescentes* complex – though some introns are maintained via vertical inheritance. While horizontal gene transfer (HGT) is considered the primary driver of fungal intron acquisition [51, 52, 57, 58], the exact mechanisms remain debated. Notably, Mayers *et al*. [58] proposed that hyphal anastomosis and cytoplasmic exchange could facilitate mitochondrial DNA transfer without nuclear recombination. The high sequence similarity of introns observed here across different *Amanita* sections and even distinct genera (Table S11) supports HGT as a plausible scenario. Although sequence data alone cannot confirm the mechanism, cytoplasmic exchange via anastomosis represents a likely route for mitogenomic flux in basidiomycetes like *Amanita*.

## Conclusion

While mitogenomes are widely utilized in other eukaryotes, their application in fungal phylogenetics remains limited. Our study demonstrates that the mitogenome provides superior phylogenomic resolution compared to traditional barcode loci, particularly in resolving species complexes. Specifically, we identified five well-supported cryptic lineages within the *A*. ‘*cruentilemurum*’ complex. Contrary to expectations, intron occurrence, intron number and gene synteny did not show a strict phylogenetic correlation across species. This highlights the need for broader sampling across the *Amanita* genus to fully trace the dynamics of mitogenomic rearrangements and identify evolutionary phenomena such as hybridization, HGT or ILS.

We underscore the utility of fungal mitochondrial data as a powerful complementary tool at both intra- and interspecific levels. Nevertheless, for formal species identification, mitogenomics should be part of an integrative approach alongside morphology, ecology and geography. Finally, while phylogenetic inference based on PCGs, *rps3* and ribosomal loci effectively clarifies the evolutionary history of these taxa, only a comparative genomics approach allows for the full recognition of the high variability in gene synteny and genomic rearrangements within *Amanita*.

## Supplementary material

10.1099/mgen.0.001792 Supplementary Material 1.

10.1099/mgen.0.001792 Supplementary Material 2.

## Abbreviations

HEG: homing endonuclease gene
HGT: horizontal gene transfer
IBD: isolation-by-distance
ILS: incomplete lineage sorting
K2P: Kimura-2-parameter
LCB: locally collinear block
ML: maximum likelihood
PCG: protein-coding gene
