# Supplementary material for: Mitogenomics in Amanita, evolution, species delimitation and its utility at the species complex level
Source: Microb Genom. 2026 Jul 30;12(7):001792. doi: 10.1099/mgen.0.001792 (PMC13424474; doi:10.1099/mgen.0.001792)
Supplement: Supplementary Material 2. [file mgen-12-01792-s002.pdf]

***Title*: Mitogenomics in Amanita, evolution, species delimitation, and its utility at the species complex level**

CHRISTIAN QUINTERO-CORRALES[1,2], and ROBERTO GARIBAY-ORIJEL[1]*

1- Departamento de Botánica, Instituto de Biología, Universidad Nacional Autónoma de México, Coyoacán, Ciudad de México, México.

2- Posgrado en Ciencias Biológicas, Universidad Nacional Autónoma de México, Coyoacán, Ciudad de México, México.

*E-mail: rgaribay@ib.unam.mx

Figures

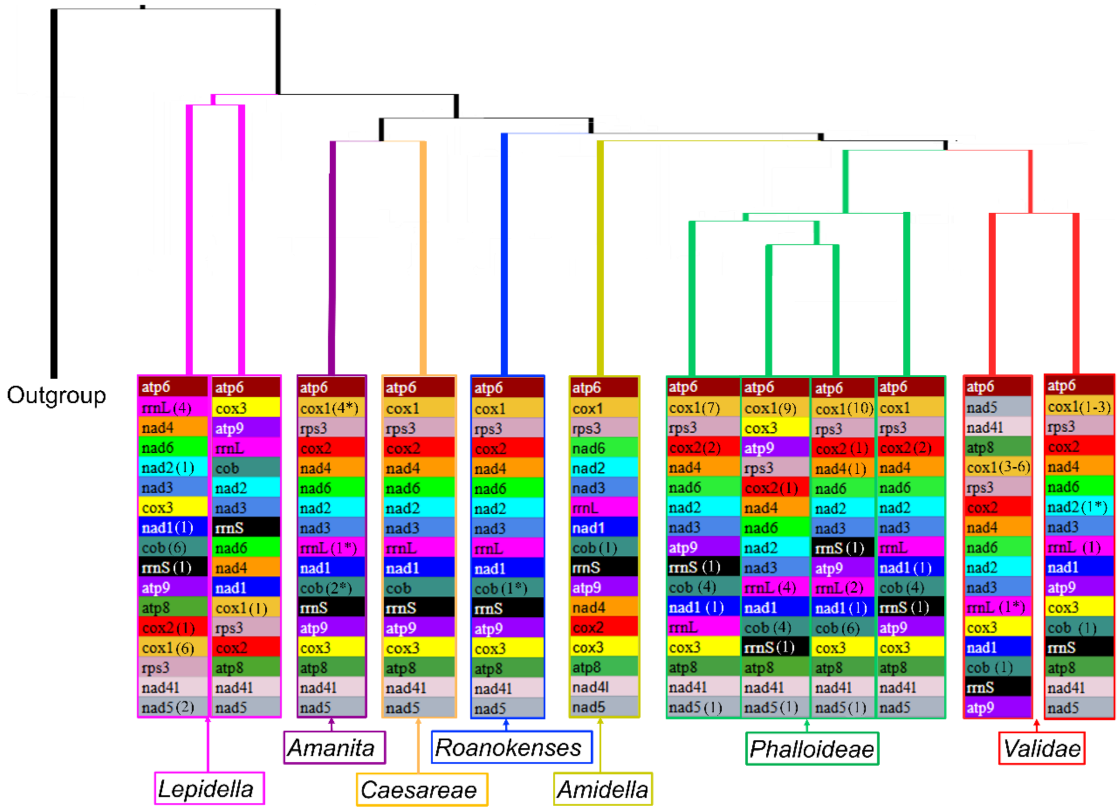

Fig. S1. *Amanita* sections phylogenetic relationships and their mitochondrial gene synteny. Species within each section with the same synteny are collapsed into one column. Each colored branch represents a different *Amanita* section (see Figure 1). Colored blocks below the tree branches show the mitochondrial gene synteny. The number inside the parentheses shows the number of introns in that specific locus. The asterisks indicate the loci that have introns only in one species of that specific section.

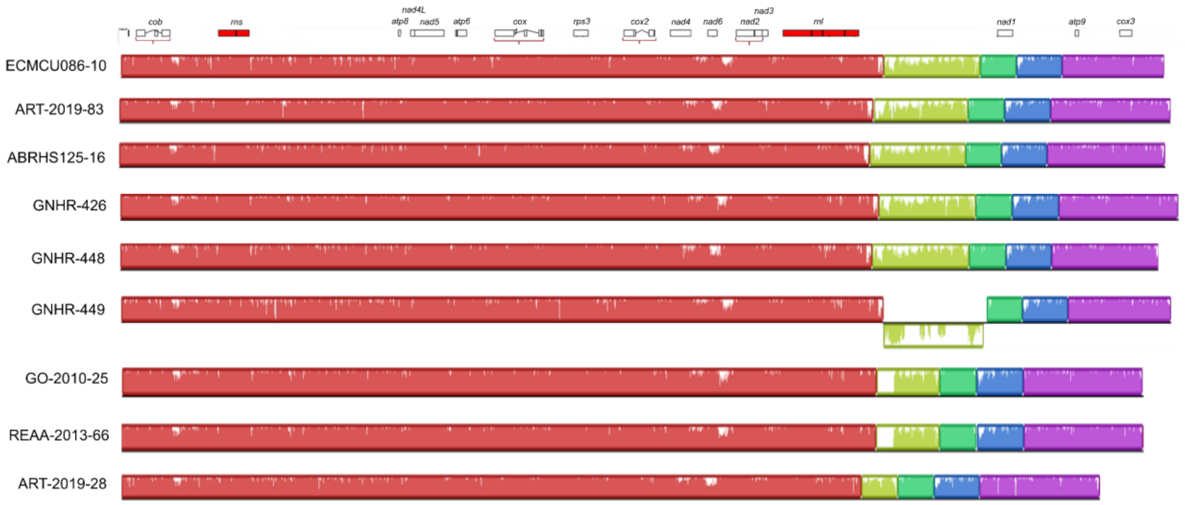

Fig. S2. Mitochondrial intra-specific variation in *A. "cruentilemurum"* samples using Mauve v2.4.0. Each square or rectangle above the image illustrates the PCG, rps3, and the two rRNA loci. The positions of each locus in the samples are marked with lines. Each colored block represents a homologous region shared among the different samples. White areas within blocks show a unique sequence for that sample.

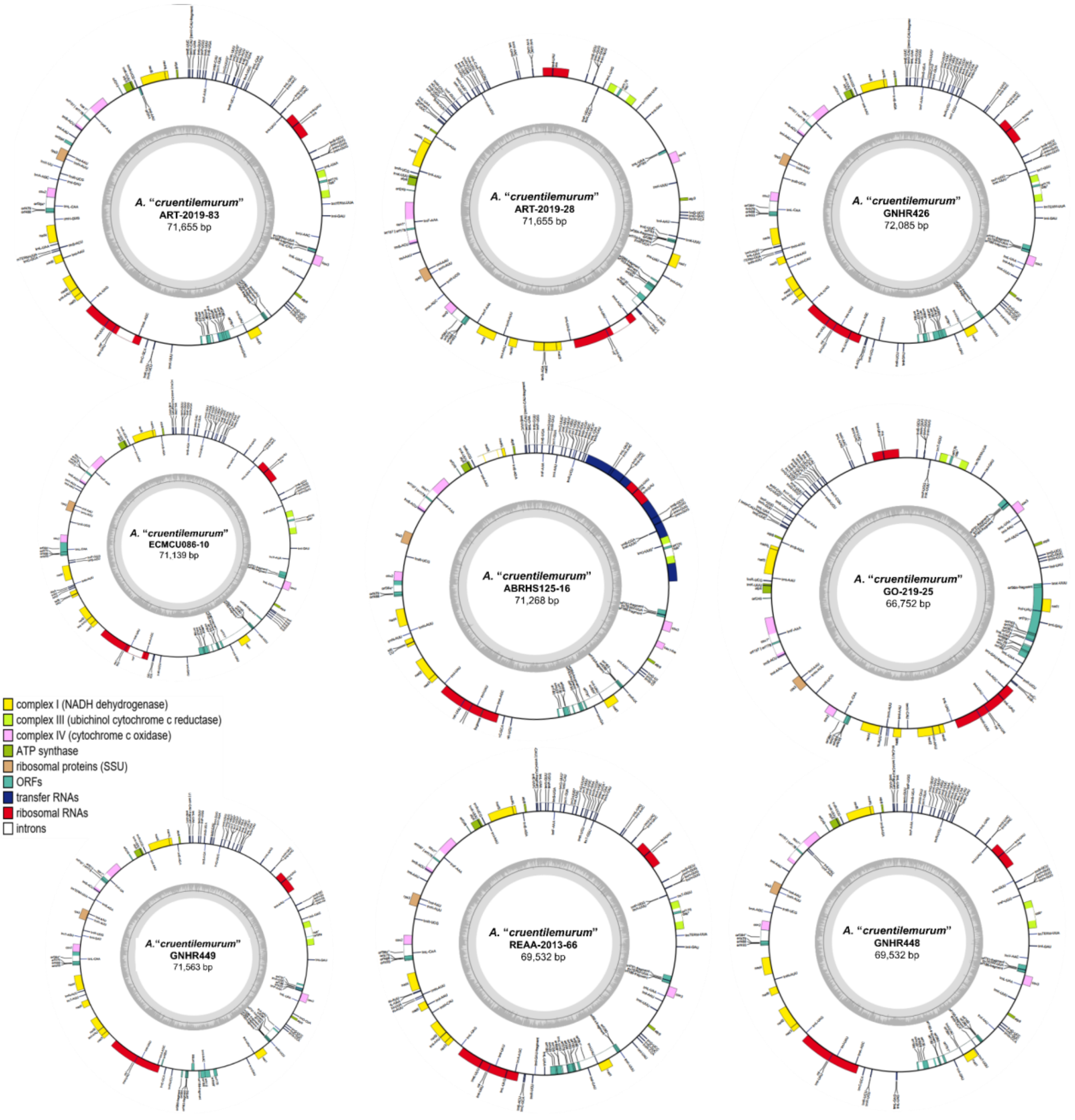

Fig. S3. Mitochondrial graphs of *A. "cruentilemurum"* samples. Different-colored blocks in the circle maps represent each gene. The blocks outside the ring are in the forward direction, while those inside are in the reverse direction.

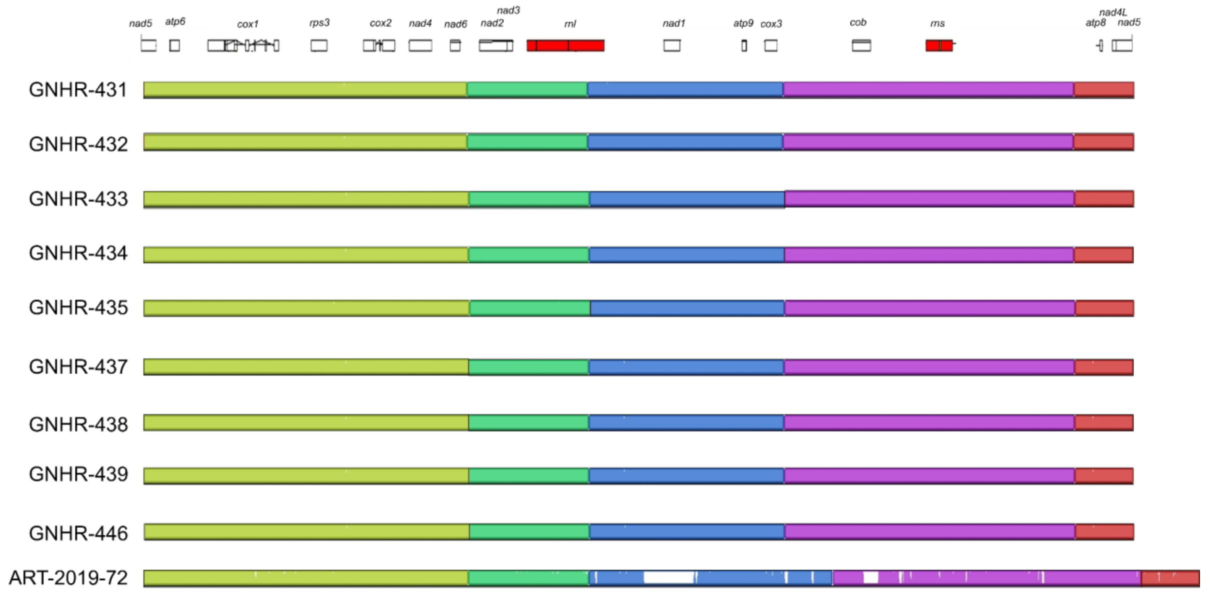

Fig. S4. Mitochondrial intra-specific variation in *A. brunneolocularis* samples using Mauve v2.4.0. Each colored block represents a homologous region shared among the different samples. White areas within blocks show a unique sequence for that sample.

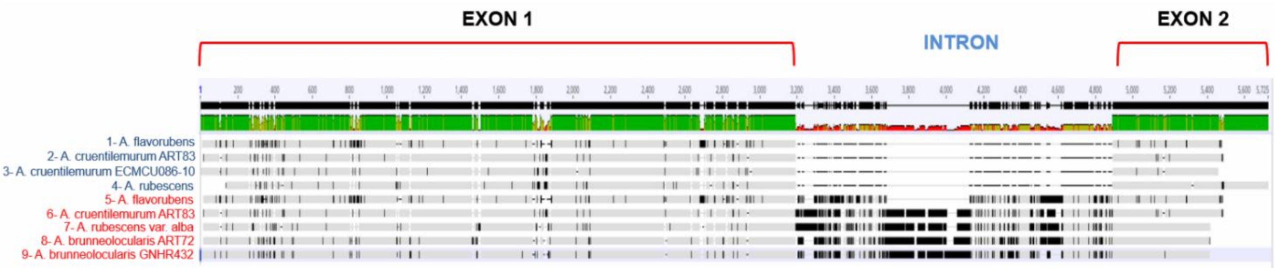

Fig. S5. Alignment of the *rnl* locus of *Rubescentes* species. The alignment was performed with MEGA using the MUSCLE algorithm of four intronless *rnl* sequences in blue (1- *A. flavorubens*, 2- *A. cruentilemurum* ART-2019-83, 3- *A. cruentilemurum* ECMCU086-10, and 4- *A. rubescens*), and five complete *rnl* sequences in red (5- *A. flavorubens*, 6- *A. cruentilemurum* ART-2019-83, 7- *A. rubescens* var. *alba*, 8- *A. brunneolocularis* ART-2019-72, 9- *A. brunneolocularis* GNHR432). The green bar indicates the consensus sequences where the alignment had a high percentage of similarity, while the short and red region shows poor similarity values. The upper part shows the region where the exons (red brackets) and introns (in blue) are.
